# The Value of Mouse Models of Rare Diseases: A Spanish Experience

**DOI:** 10.3389/fgene.2020.583932

**Published:** 2020-10-14

**Authors:** Silvia Murillo-Cuesta, Rafael Artuch, Fernando Asensio, Pedro de la Villa, Mara Dierssen, Jose Antonio Enríquez, Cristina Fillat, Stéphane Fourcade, Borja Ibáñez, Lluis Montoliu, Eduardo Oliver, Aurora Pujol, Eduardo Salido, Mario Vallejo, Isabel Varela-Nieto

**Affiliations:** ^1^Biomedical Research Networking Center on Rare Diseases (CIBERER), Institute of Health Carlos III, Madrid, Spain; ^2^Instituto de Investigaciones Biomédicas Alberto Sols (IIBM), Consejo Superior de Investigaciones Científicas/Universidad Autónoma de Madrid, Madrid, Spain; ^3^Hospital La Paz Institute for Health Research (IdiPAZ), Madrid, Spain; ^4^Institut de Recerca Sant Joan de Déu (IRSJD), Barcelona, Spain; ^5^Gregorio Marañón Institute for Health Research (IISGM), Madrid, Spain; ^6^Faculty of Medicine, University of Alcalá (UAH), Alcalá de Henares, Spain; ^7^Centre for Genomic Regulation (CRG), Barcelona Institute of Science and Technology (BIST), Barcelona, Spain; ^8^Universitat Pompeu Fabra (UPF), Barcelona, Spain; ^9^Spanish National Center for Cardiovascular Research (CNIC), Institute of Health Carlos III, Madrid, Spain; ^10^Biomedical Research Networking Center on Frailty and Healthy Ageing (CIBERFES), Institute of Health Carlos III, Madrid, Spain; ^11^Institut d’Investigacions Biomèdiques August Pi i Sunyer (IDIBAPS), Barcelona, Spain; ^12^Bellvitge Biomedical Research Institute (IDIBELL), L’Hospitalet de Llobregat, Barcelona, Spain; ^13^Biomedical Research Networking Center on Cardiovascular Diseases (CIBERCV), Institute of Health Carlos III, Madrid, Spain; ^14^Cardiology Department, Fundación Jiménez Díaz University Hospital Health Research Institute (IIS-FJD), Madrid, Spain; ^15^National Center for Biotechnology (CNB), Spanish National Research Council, Madrid, Spain; ^16^Catalan Institution of Research and Advanced Studies (ICREA), Barcelona, Spain; ^17^Unidad de Investigación, Hospital Universitario de Canarias, Instituto de Tecnologías Biomédicas (ITB), La Laguna, Spain; ^18^Biomedical Research Networking Center on Diabetes and Metabolic Diseases (CIBERDEM), Institute of Health Carlos III, Madrid, Spain

**Keywords:** orphan diseases, animal models, preclinical research, novel therapies, ethics, transparency

## Abstract

Animal models are invaluable for biomedical research, especially in the context of rare diseases, which have a very low prevalence and are often complex. Concretely mouse models provide key information on rare disease mechanisms and therapeutic strategies that cannot be obtained by using only alternative methods, and greatly contribute to accelerate the development of new therapeutic options for rare diseases. Despite this, the use of experimental animals remains controversial. The combination of respectful management, ethical laws and transparency regarding animal experimentation contributes to improve society’s opinion about biomedical research and positively impacts on research quality, which eventually also benefits patients. Here we present examples of current advances in preclinical research in rare diseases using mouse models, together with our perspective on future directions and challenges.

## Introduction

Animal research has contributed greatly to advance human health and quality of life. The use of laboratory animals increased exponentially in the 20th century and they are currently employed in almost every field of biomedical research. Animal models reproduce many aspects of human biological and pathological processes, and provide key information on the molecular pathophysiology of human diseases. Non-animal approaches based mainly on cell or tissue/organ culture, and computational methods like data mining/generation, may help to predict clinical outcomes and reduce animal use (Cronin, 2016), but they cannot mimic the complexity of human biology. Animals remain the best model–however imperfect–to predict and characterize disease activity in patients (Garattini and Grignaschi, 2017).

Choosing a suitable animal model is a critical step in basic and preclinical research, and is usually based on a number of criteria, including species similarities to humans (the greater the phylogenetic closeness, the more similar is the genetic composition, anatomy, and physiology), genetic homogeneity, previous knowledge of the model, cost, availability, translatability of the results, ease of manipulation, and ethical implications, among others (National Research Council, 1998). Thus, the laboratory mouse is the most widely used mammalian animal in biomedical research, representing more than 60% of the total number of animals used in the EU (European Commission, 2019).

Genome manipulation and phenotype characterization is a common strategy for studying human pathology in animals, and particularly, in mice. In the last few years, CRISPR-Cas9-based genome editing has transformed the field and greatly expanded the repertoire of animal/cell systems available for disease modeling (Ahmad and Amiji, 2018). Gene homology between mouse and man is an essential prerequisite for pursuing this genotype-to-phenotype approach. Homogenization of the genetic background by inbreeding is also an important factor to reduce experimental variability. In this context, the International Mouse Phenotyping Consortium has generated, phenotyped and archived more than 6000 knockout mice on the C57BL/6 background, the most well-known and widely used inbred mouse strain (Cacheiro et al., 2019). Complete catalogs of genetically modified mouse models are available online at the International Phenotype Mouse Consortium and The Jackson Laboratory webpages (Table 1).

**TABLE 1:** Reference online resources on RDs, mouse models, legislation, and recommendations on animal experimentation.

Online resource	Description	URL
International Rare Diseases Research Consortium (IRDiRC)	International consortium of national and international governmental and non-profit funding bodies, companies, umbrella patient advocacy organizations, and scientific researchers to accelerate diagnosis and contribute to the development of new therapies for RDs	https://irdirc.org/
Orphanet	European website providing information about orphan drugs and rare diseases. It contains content both for physicians and for patients	https://www.orpha.net
Orphadata	Comprehensive, quality data sets related to RDs and orphan drugs from the Orphanet knowledge base, in reusable formats.	http://www.orphadata.org
Biomedical Research Center Network for RDs (CIBERER)	Spanish network cooperative structure of basic and clinical research groups with the purpose of (1) generating new scientific knowledge on the causes and mechanisms of RDs, and (2) developing new treatments and diagnostic procedures for these illnesses.	https://www.ciberer.es/en
Committee for Orphan Medicinal Products (COMP)	Committee of the European Medicines Agency (EMA) responsible for recommending orphan designation of medicines for rare diseases.	https://www.ema.europa.eu/en/committees/committee-orphan-medicinal-products-comp
The Jackson Laboratory	Independent, non-profit organization focusing on mammalian genetics research to advance human health	https://www.jax.org
Jackson Laboratory Rare and Orphan Disease Center	Jackson Lab center focused in the generation of mouse models for rare disease research.	https://www.jax.org/research-and-faculty/research-centers/rare-and-orphan-disease-center
International Phenotype Mouse Consortium	International consortium of research institutions to identify the function of every protein-coding gene in the mouse genome.	https://www.mousephenotype.org
International Mouse Phenotyping Resource of Standardized Screens (IMPReSS)	Standardized phenotyping protocols which are essential for the characterization of mouse phenotypes.	https://www.mousephenotype.org/impress/
European Commission	European legislation for the protection of animals used for scientific purpose 2010/63/EU directive	https://ec.europa.eu/environment/chemicals/lab_animals/legislation_en.htm http://data.europa.eu/eli/dir/2010/63/oj
Animal Research Reporting of *In Vivo* Experiments (ARRIVE) guidelines	Gold Standard publication Checklist reporting Guidelines	https://arriveguidelines.org/
European Quality in Preclinical Data (EQIPD) Consortium		https://quality-preclinical-data.eu/

## Animal Models for Rare Disease Research

The definition of a rare disease (RD) in Europe is a disease with a prevalence of <1 in 2000, whereas *ultra*-RDs affect <1 in 50,000. RDs comprise more than 7000 different conditions (Orphadata, Table 1), usually severe, clinically complex and chronic, affecting 3.5–5.9% of the world’s population (Nguengang Wakap et al., 2020), most of whom are children. The fact that each RD affects a relatively small number of patients has resulted in limited knowledge of RDs at the clinical level, which often delays an early and accurate diagnosis–patients can wait 8 years before receiving a diagnosis–and a potential therapy. Alarmingly, 95% of RDs have no approved or effective treatments, in part because RDs are underserved by pharmaceutical companies. Accordingly, RDs are considered a public health priority and specific research programs as the International Rare Diseases Research Consortium (IRDiRC) (Table 1) have been established to foster knowledge development (Gahl et al., 2016; European Commission, 2017).

Animal models are indispensable to identify the genetic bases and molecular mechanisms of RDs, as well as to understand their physiopathology, clinical heterogeneity and genotype-phenotype correlations. Indeed, RDs are excellent candidates for animal models, particularly in the context of genetically modified mice, as most RDs involve mutations in a single gene (Institute of Medicine, 2010). Due to the scarcity of available information on RD models, however, one of the major issues hindering translational research is the (incorrect) choice of model in preclinical studies. To address this and other issues some initiatives have been recently launched to generate and register RD mouse models. For instance, the Jackson Laboratory Rare and Orphan Disease Center (Table 1) has generated animal models for Friedreich’s ataxia, Rett syndrome and spinal muscular atrophy. Likewise, the Infrafrontier platform provides access to 670 mouse strains that are related to nearly 1200 distinct RDs. Information about RD mouse models can also be obtained from the governmental agencies responsible for the evaluation of orphan medicinal product designation applications from pharmaceutical companies. In this context, Vaquer et al. (2013) compiled a list of 57 mammalian animal models for metabolic, neuromuscular, and ophthalmological orphan-designated conditions, based on information gathered by the European Medicines Agency (EMA). Additionally, some countries have developed specific national plans to prioritize RD research. For example, in Spain, the Biomedical Research Center Network for RDs (CIBERER) of the Carlos III Health Institute has contributed to the advancement of RD research by (i) developing new animal models, (ii) performing preclinical assays of novel therapeutics, and (iii) creating a mouse model phenotyping unit and a working group to register model information.

Here, we discuss some representative examples of RD mouse models under investigation at CIBERER (Table 2), which serve to illustrate the phenotypic variability of RDs and the possibilities offered by animal modeling to fill the knowledge gaps regarding in this area, and to contribute to the IRDiRC’s goal of accelerating diagnosis and approving 1000 new therapies for RDs by 2027.

**TABLE 2:** Rare disease mouse models.

ORPHA number	Allelic symbol	Allele name	Genotype	MGI number	References
**Adrenoleukodystrophies**					
43	*Abcd2*^*tm1Apuj*^	ATP-binding cassette, sub-family D (ALD), member 2; targeted mutation 1, Aurora Pujol	Homozygous	3617308	Pujol et al., 2002, 2004; Fourcade et al., 2008; Lopez-Erauskin et al., 2011, 2012; Schluter et al., 2012; Ruiz et al., 2015
	*Abcd1*^*tm1Kds*^	ATP-binding cassette, sub-family D (ALD), member 1; targeted mutation 1, Kirby D Smith	Homozygous	2446588,2680904	
**Rare aminoacidurias and hyperoxalurias**					
2195	*Slc7a8*^*tm1Gen*^	Solute carrier family 7 (cationic amino acid transporter, y+ system), member 8; targeted mutation 1, Genoway	Homozygous	6323258,6323255	Vilches et al., 2018
			Heterozygous	6323256	
1032	*Slc16a10*^*m1Ingm*^	Solute carrier family 16 (monocarboxylic acid transporters), member 10; mutation 1, Ingenium Pharmaceuticals	Homozygous	5544309	
93598	*Agxt*^*tm1Ull*^	Alanine-glyoxylate aminotransferase; targeted mutation 1, Eduardo C Salido	Homozygous	3717654,5314652	Salido et al., 2006; Knight et al., 2012
93600	*Grhpr*^*Gt(OST383093)Lex*^	Glyoxylate reductase/hydroxypyruvate reductase; gene trap OST383093, Lexicon Genetics.	Homozygous	5314653	Knight et al., 2012
**Rare cardiomiopathies**					
247	*AAV-PCSK9^*DY*^ ApoE^–/–^ AAV-PCSK9^*DY*^*	AAV-based vector for targeted transfer of the *PCSK9(DY)* gene			Cruz et al., 2015; Roche-Molina et al., 2015
**Rare deafness**					
90635	*Tecta*^*tm3.1Gpr*^	Tectorin alpha; targeted mutation 3.1, Guy P Richardson	Homozygous	5527172	Legan et al., 2014
			Heterozygous	5527171	
90635	*Tecta*^*tm4.1Gpr*^	Tectorin alpha; targeted mutation 4.1, Guy P Richardson	Homozygous	5527174	Legan et al., 2014
			Heterozygous	5527173	
90635	*Tecta*^*tm5.1Gpr*^	Tectorin alpha; targeted mutation 5.1, Guy P Richardson	Homozygous	5527176	Legan et al., 2014
			Heterozygous	5527175	
90636	*Gjb2*^*tm1Ugds*^ *Tg(Otog-cre)1Ugds*	Gap junction protein, beta 2; targeted mutation 1, Unite de Genetique des Deficits Sensoriels	Homozygous conditional	3588875	Cohen-Salmon et al., 2002
	*Gjb2*^*tm1Ugds*^ *Tg(Sox10-cre)1Wdr*		Homozygous conditional	5571190	Takada et al., 2014
9063	*Mpzl2*^*tm1.1Jczp*^	Myelin protein zero-like 2; targeted mutation 1.1, Juan Carlos Zuniga-Pflucker	Homozygous	6358214	Wesdorp et al., 2018
73272	*Igf1*^*tm1Arge*^	Insulin-like growth factor 1; targeted mutation 1, Argiris Efstratiadis	Homozygous	3688508	Liu et al., 1993; Camarero et al., 2001, 2002; Cediel et al., 2006; Fuentes-Santamaria et al., 2016, 2019
**Albinism**					
79431	*Tg(Tyr-Th,-Gch1)6775 Lmon*	Transgene insertion 6775, Lluis Montoliu		4443311 (EM: 02610)	Lavado et al., 2006; Murillo-Cuesta et al., 2010
79431	*Tg(Tyr)1999 Lmon*	Transgene insertion 1999, Lluis Montoliu		5787939 (EM: 03096)	Lavado et al., 2006; Murillo-Cuesta et al., 2010

*Representative examples of mouse models developed or studied in the CIBER consortium to increase knowledge, provide diagnosis, and explore advanced therapies in RDs, identified by their ORPHA number. Rare Disease Database at Orphanet website (https://www.orphanet.com) and Mouse Genome Database (MGD) at the Mouse Genome Informatics (MGI) website, The Jackson Laboratory, Bar Harbor, Maine (http://www.informatics.jax.org) (June 2020).*

### Metabolic RDs

They encompass a large and heterogeneous group of RDs caused by mutations affecting the function of enzymes, transporters, receptors, or hormones involved in metabolizing and transporting small (e.g., amino acids or neurotransmitters) or complex (i.e., glycogens or lipids) molecules, and defects in mitochondrial energy metabolism. One of the most extensively investigated is phenylketonuria, which severely affects the brain by interfering with dopamine and serotonin metabolism (Winn et al., 2018). *Pah^*enu*2/enu2^* and *Pah^*enu*3/enu3^* mice mimic human phenylketonuria pathophysiology and have aided in discovering mechanisms and therapies based on phenylalanine-restricted diets (Winn et al., 2018). Similarly, aromatic amino acid decarboxylase deficiency is a defect in dopamine and serotonin synthesis that also causes devastating central nervous system degeneration. *Ddc^TM 1^.^1*Nwlh*^* mutant mice have been used to study the disease (Lee et al., 2013) and to evaluate adeno-associated viral gene therapy, which improved both survival and brain levels of dopamine and serotonin (Lee et al., 2016). A clinical trial using this approach is ongoing with encouraging results (Chien et al., 2017).

X-linked adrenoleukodystrophy (X-ALD) is another severe neurometabolic disease characterized by progressive central demyelination, adrenal insufficiency and accumulation of saturated very long-chain fatty acids, and caused by loss of function of the ABCD1 peroxisomal transporter (Ferrer et al., 2010). To date, no pharmacological treatment has been proven to be beneficial and current therapeutic options are unsatisfactory and restricted to bone marrow transplants and hematopoietic stem cell gene therapy, but most patients remain untreated. Mouse models uncovered the factors that account for genotype-phenotype correlation in human disease variants. The *Abcd1*^–^ mutant mouse exhibits late-onset axonal degeneration of the spinal cord corticospinal tracts and microglial and astroglial activation, compatible with chronic low-level stimulation of the innate immune response, and constitutes a good model for ALD (Pujol et al., 2002; Ruiz et al., 2015). The *Abcd2* gene product shares physiological and biochemical functions with that of *Abcd1* (Pujol et al., 2004), and the *Abcd1^–^/Abcd2^–/–^* double mutant presents with an earlier and more severe axonal degenerative phenotype, constituting a more useful model for preclinical evaluation (Pujol et al., 2004). These mouse models revealed that X-ALD shares pathogenic processes with other neurodegenerative disorders (Galea et al., 2012), including redox dyshomeostasis, mitochondrial dysfunction, and proteostasis malfunction (Fourcade et al., 2015). Encouraging preclinical results with neurotrophic factors and antioxidants (Pujol, 2016) have paved the way for the launch of three phase II/III clinical trials for ALD (Casasnovas et al., 2019), and the approval of two orphan drug designations.

Defects in glyoxylate and hydroxyproline hepatic metabolism result in the hepatic overproduction of oxalate and primary hyperoxaluria (PH) – an *ultra*-RD with a prevalence of 1–3 in 10^6^ individuals (Cochat and Rumsby, 2013). PH1, the most common and severe form, is caused by *AGXT* mutations (Milliner et al., 1993), whereas PH2 and PH3 are caused by mutations in *GRHPR* and *HOGA1*, respectively. Loss of function mutations in any of these genes results in impaired detoxification of glyoxylate, which is converted into oxalate. PH patients present elevated oxalate concentrations in plasma and urine, oxalate deposition in multiple organs, recurrent kidney stone episodes and chronic renal failure, which results in end-stage renal disease. Current therapies include large daily fluid intake and medications to reduce oxalate production (Cochat et al., 2012), but they do not eliminate recurring stones and renal disease. Combined liver and kidney transplantation is the only curative treatment available, but is associated with significant morbi-mortality and problems related to donor organ shortage and life-long immunosuppressive treatment.

The *Agxt^TM 1*Ull*^* mouse reproduces the main PH1 features (Salido et al., 2006) and has been used to evaluate promising experimental therapies (Martin-Higueras et al., 2017). Regulation of oxalate transepithelial flux in the gut following intestinal colonization with *Oxalobacter* (Hatch et al., 2011) has received an innovative new drug designation by the United States Food and Drug Administration and is in clinical trials. Similarly, gene therapy with adeno-associated vectors carrying human *AGXT* under the control of a liver-specific promoter achieved a long-term metabolic correction (Salido et al., 2011), and was granted an EMA orphan drug designation. Deletion of the glycolate oxidase gene, inhibition of its enzymatic product or suppressing its expression with short-interfering RNA (siRNA) resulted in a substantial reversal of the hyperoxaluric phenotype (Martin-Higueras et al., 2016), the latter is currently being evaluated in a clinical trial with encouraging preliminary results. Therapies based on *in vivo* CRISPR-Cas9 technology are also a potential strategy for curing PH1 by substrate reduction with the administration of AAV-mediated glycolate oxidase-targeted guide RNAs (Zabaleta et al., 2018). We have generated a *Grhpr* knockout mouse for PH2 (Knight et al., 2012), and both *Agxt* and *Grhpr* mutant mice have been used to test the potential of inhibiting hepatic lactate dehydrogenase with siRNA to treat PH (Lai et al., 2018), which has moved to a clinical trial. In contrast to the models for PH1 and PH2, the mouse model for PH3 generated by the International Knockout Mouse Consortium (*Hoga1^TM 2*a(KOMP)Wtsi*^*, MGI:4419886) does not have the expected phenotype and it is currently being used to investigate differences in mouse and human glyoxylate metabolism. This example highlights a key point, which is that the understanding of the differences in the metabolic interactome between species is fundamental for the efficient transfer of the knowledge from experimental models to clinical practice.

Rare aminoacidurias caused by defects in amino acid transporters are being studied with murine models, which emerge as a promising tool to design evidence-based therapies to halt the progression of the disease. Using the *Slc16a10^–/–^ Slc7a8^–/–^* mouse and a targeted metabolomics approach, it was confirmed that both transporters functionally cooperate *in vivo*. This approach also uncovered compensation mechanisms that explain the lack of human basolateral neutral aminoacidurias (Vilches et al., 2018). Similarly, the *Slc7a7^–/–^* model of lysinuric protein intolerance resembles the human phenotype, including malabsorption and impaired reabsorption of cationic amino acids, hypoargininemia, and hyperammonemia, and importantly, responses to citrulline treatment, which improved the metabolic derangement and survival (Bodoy et al., 2019).

### Rare Cardiac Diseases

Arrhythmogenic right ventricular cardiomyopathy is a severe disease characterized by ventricular fibrofatty replacement of cardiomyocytes, contractile defects, and high risk for developing malignant arrhythmias, which can ultimately lead to sudden cardiac death especially in young athletes (Gandjbakhch et al., 2018). More than 50% of the 380 mutations identified lie within *PKP2*, which encodes the desmosomal protein plakoglobin-2, a major component of cell-to-cell junctions (van Tintelen et al., 2006). Given the complexity of developing multiple transgenic animals, a novel approach was developed by delivering genes encoding mutated proteins into wild-type mice using adeno-associated viruses (Roche-Molina et al., 2015). Using this strategy, C57BL6/J mice stably expressed the R735X version of *PKP2*, a dominant-negative mutant, driven by a cardiac-specific promoter, resulting in development of an arrhythmogenic right ventricular cardiomyopathy phenotype following exercise (Cruz et al., 2015). Although no evidence of myocardial fibrosis or fibrofatty cardiomyocyte replacement was observed, a miss localization of the gap-junction protein connexion-43 was evident. This model provides a versatile and accessible tool for investigating this devastating disease.

### Albinism

Murine models have been central to understand this rare genetic condition primarily associated with severe visual deficits and variable hypopigmentation, and caused by mutations in at least twenty genes (Montoliu and Marks, 2017). Vision and hearing deficits have been characterized in the *Tyr* mutant mouse, a model for human oculocutaneous albinism 1 (Lavado et al., 2006; Murillo-Cuesta et al., 2010). Additional mouse models have been generated using CRISPR-Cas9 tools, including those addressing the role of non-coding DNA of regulatory elements in *Tyr* gene expression (Seruggia et al., 2015).

### Sensorineural Hearing Loss

Approximately half of all cases of both non-syndromic and syndromic human hearing loss (HL) are due to rare mutations. TECTA-based human deafness is an example of autosomal dominant non-syndromic HL, in which domain-specific alterations in the glycoprotein Tecta, leading to changes in the tectorial membrane of the cochlea, have been studied using *Tecta* mutant mice (Legan et al., 2014). Autosomal recessive non-syndromic HL, which in the majority of cases is caused by mutations in *GJB2* and *GJB6*, encoding the gap junction proteins connexin 26 and 30, respectively, has been studied using conditional mutant mice. Thus, *Gjb2^TM 1*Ugds*^* mouse shows a decrease in *Cx26* expression, extensive loss of cochlear epithelial cells and an increase in hearing thresholds (Cohen-Salmon et al., 2002; Crispino et al., 2017).

Syndromic HL is a common condition in many RDs including insulin-like growth factor I (IGF-1) deficiency, an *ultra*-RD caused by homozygous mutations in *IGF1* and associated with growth retardation, intellectual deficit, and HL (Varela-Nieto et al., 2013). The use of experimental models is practically the only way to investigate the pathology of *ultra*-RDs. In this respect, the *Igf1^TM 1*Arge/tm*1*Arge*^* mouse (Liu et al., 1993) recapitulates the human phenotype, and presents with severe deafness, neural loss (Cediel et al., 2006) and alterations in the auditory central pathway (Fuentes-Santamaria et al., 2016; Fuentes-Santamaria et al., 2019), offering a unique window into the role of the IGF-1 in human hearing.

## Discussion

Animal experimentation is essential for understanding the pathogenic mechanisms of RDs and developing new, safe and effective treatments (Garattini and Grignaschi, 2017). This is especially true for RDs whose low prevalence is associated with a lack of knowledge, delays in diagnosis, and absence of effective treatments in most cases (European Commission, 2017). Non-animal experimental approaches provide valuable information, but they are far from reproducing the complexities and interactions in a living organism (Cronin, 2016). Rather than an alternative, non-animal methods are a useful complementary approach that helps to reduce the number of specimens used in biomedical research (European Commission, 2018).

The mouse is currently the most commonly used species due to its genetic tractability, relative ease of genome editing and cost-efficient management (European Commission, 2019). During the last 20 years, public and private initiatives have made a strong effort to generate and phenotype many hundreds of genetically modified strains (Cacheiro et al., 2019). However, it has been only recently that special attention has been paid to RDs (Institute of Medicine, 2010; Gahl et al., 2016). Information on RD mouse models is limited and scattered across different databases, which could hamper the preclinical testing of new therapeutic approaches. It would be useful to gather all the data from already existing mutant mouse databases with those from the orphan drug evaluation committees in international agencies (Vaquer et al., 2013) and from national initiatives for RDs research. As an example, the Spanish CIBERER consortium has generated mouse models for some RDs that have been shown to be effective for preclinical testing of new drugs (Table 2).

The usefulness of mouse models to advance RD research should not make us forget the importance of the ethical aspects and transparency in animal research. The use of animals in biomedical research remains a contentious issue in society (Matthews, 2008). Citizens demand treatments that require preclinical safety and efficacy testing, but they are increasingly concerned by animal welfare and demand the elimination of pain, and ultimately, of animal experimentation. Authorities and the scientific community are devoted to protect public health and the environment, and require the testing of new medicines, chemicals, and food products in animal models. But they are also fully committed to animal welfare and to the progressive reduction of animal testing (European Commission, 2018), as stated in the current legislation. There is a large body of laws and regulations regarding the use of animals for scientific research and educational purposes. The 2010/63/EU directive (Table 1) states that: (i) animal experimentation can be carried out only after a number of independent evaluations, and authorization from the competent authority; (ii) researchers must reasonably justify the use of experimental animals over alternative methods; (iii) experiments involving animals can only be conducted by competent and experienced professionals in authorized facilities; (iv) the 3Rs principle (reduction, refinement, and replacement) has always to be considered (Mocho, 2020). However, it is critical to improve communication with the general public to convey the fact that animal experimentation is necessary not only to protect human health, but also to protect animals and the environment.

Society also demands transparency regarding animal experimentation. Modern science is now so complex that citizens are often unaware of the gaps in knowledge still existing and wrongly assume that the use of animals is no longer necessary. It is essential that researchers take a stand and clearly explain their position with regard to the use of animals (Van Zutphen, 2002). To fill these gaps, some initiatives have arisen from scientific organizations addressing the requirement for transparency (Montoliu, 2018). The scientific community hopes that society will soon better understand the benefits of the use of animals in research and will provide greater support for animal experimentation, resolving the current controversies. In addition, initiatives like the Animal Research Reporting of *In Vivo* Experiments (ARRIVE) guidelines and the European Quality in Preclinical Data (EQIPD) have arose to solve challenges with regard to the robustness, rigor, and validity of research data, which often impact the transition from preclinical to clinical testing.

Ethics and transparency in this context will undoubtedly enhance the quality of biomedical research and societal engagement (Van Zutphen, 2002; Montoliu, 2018).

## Author Contributions

SM-C, IV-N, RA, LM, SF, AP, BI, EO, and ES wrote the manuscript. All the co-authors revised and approved the manuscript.

## Conflict of Interest

The authors declare that the research was conducted in the absence of any commercial or financial relationships that could be construed as a potential conflict of interest.
